# Automated localization and segmentation of cervical lymph nodes on contrast-enhanced CT using a 3D foveal fully convolutional neural network

**DOI:** 10.1186/s41747-023-00360-x

**Published:** 2023-07-28

**Authors:** Miriam Rinneburger, Heike Carolus, Andra-Iza Iuga, Mathilda Weisthoff, Simon Lennartz, Nils Große Hokamp, Liliana Caldeira, Rahil Shahzad, David Maintz, Fabian Christopher Laqua, Bettina Baeßler, Tobias Klinder, Thorsten Persigehl

**Affiliations:** 1 Institute of Diagnostic and Interventional Radiology, Faculty of Medicine and University Hospital Cologne, University of Cologne, Cologne, Germany; 2 Philips Research, Hamburg, Germany; 3 Innovative Technologies, Philips Healthcare, Aachen, Germany; 4 Institute of Diagnostic and Interventional Radiology, University Hospital Würzburg, Würzburg, Germany

**Keywords:** Artificial intelligence, Deep learning, Lymph nodes, Neoplasm staging, Tomography (x-ray computed)

## Abstract

**Background:**

In the management of cancer patients, determination of TNM status is essential for treatment decision-making and therefore closely linked to clinical outcome and survival. Here, we developed a tool for automatic three-dimensional (3D) localization and segmentation of cervical lymph nodes (LNs) on contrast-enhanced computed tomography (CECT) examinations.

**Methods:**

In this IRB-approved retrospective single-center study, 187 CECT examinations of the head and neck region from patients with various primary diseases were collected from our local database, and 3656 LNs (19.5 ± 14.9 LNs/CECT, mean ± standard deviation) with a short-axis diameter (SAD) ≥ 5 mm were segmented manually by expert physicians. With these data, we trained an independent fully convolutional neural network based on 3D foveal patches. Testing was performed on 30 independent CECTs with 925 segmented LNs with an SAD ≥ 5 mm.

**Results:**

In total, 4,581 LNs were segmented in 217 CECTs. The model achieved an average localization rate (LR), *i.e.*, percentage of localized LNs/CECT, of 78.0% in the validation dataset. In the test dataset, average LR was 81.1% with a mean Dice coefficient of 0.71. For enlarged LNs with a SAD ≥ 10 mm, LR was 96.2%. In the test dataset, the false-positive rate was 2.4 LNs/CECT.

**Conclusions:**

Our trained AI model demonstrated a good overall performance in the consistent automatic localization and 3D segmentation of physiological and metastatic cervical LNs with a SAD ≥ 5 mm on CECTs. This could aid clinical localization and automatic 3D segmentation, which can benefit clinical care and radiomics research.

**Relevance statement:**

Our AI model is a time-saving tool for 3D segmentation of cervical lymph nodes on contrast-enhanced CT scans and serves as a solid base for N staging in clinical practice and further radiomics research.

**Key points:**

• Determination of N status in TNM staging is essential for therapy planning in oncology.

• Segmenting cervical lymph nodes manually is highly time-consuming in clinical practice.

• Our model provides a robust, automated 3D segmentation of cervical lymph nodes.

• It achieves a high accuracy for localization especially of enlarged lymph nodes.

• These segmentations should assist clinical care and radiomics research.

**Graphical Abstract:**

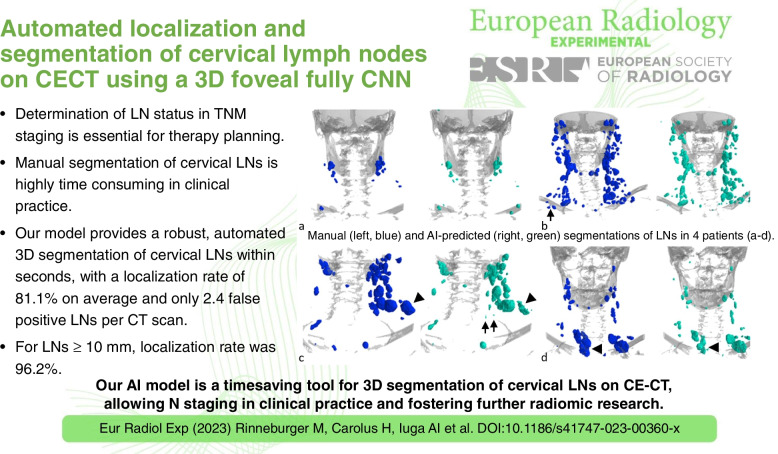

**Supplementary Information:**

The online version contains supplementary material available at 10.1186/s41747-023-00360-x.

## Background

In oncology, the determination of lymph node (LN) involvement is crucial for assessing treatment options and consequently directly impacts the patient’s outcome and overall survival [1]. Nodal involvement is often diagnosed based on computed tomography (CT) imaging, positron emission tomography or magnetic resonance imaging scans, sonography, and when appropriate biopsy confirmation [2, 3]. In cancers of the head and neck, confirmation of N0 staging status is especially crucial in decision-making for elective neck dissection [4]. While neck dissection can on the one hand prolong overall survival in various tumor entities, on the other hand, it can result in substantial morbidity, thus impairing the patient’s quality of life. Moreover, concerning radiation therapy, the second-line treatment for locoregional control, the dosage is applied according to the likelihood of malignancy of LNs and adapted according to changes in size throughout the course of therapy [5].

In clinical practice, nodal status is evaluated through imaging and biopsy, including measurement of the short-axis diameter (SAD) of LNs on axial contrast-enhanced CT (CECT) scans. For initial tumor staging at the time of diagnosis and subsequent restaging, different standardized diagnostic criteria are applied depending on the tumor entity. While solid tumors are typically assessed using the revised response evaluation criteria in solid tumors (RECIST) version 1.1 [6], for lymphomas, modified evaluation criteria such as the Lugano criteria [7] and the response evaluation criteria in lymphoma (RECIL) [8] have been proposed in recent years, emphasizing on bilateral rather than unilateral LN measurements.

LNs with an SAD ≥ 10 mm on axial CT or magnetic resonance imaging scans as well as LNs with other radiologically detectable worrisome features such as necrosis are regarded as highly suspicious, as recently summarized in the Lymph Node Reporting and Data System (Node-RADS) version 1.0 [9–11]. However, especially in head and neck cancer and melanoma, microscopic metastases may also be found in non-enlarged LNs [12, 13], so that it is also important to monitor the size of smaller LNs over time. Moreover, it has been reported that in patients with oral squamous cell carcinoma, overall LN volume, including enlarged and non-enlarged LNs, seems to be a risk factor for locoregional recurrence independent of pathological N classification [14]. These findings point to the fact that imaging as a sole basis for defining N status has significant limitations, and additional ultrasound-guided fine needle aspiration and sentinel node biopsy should be considered to increase sensitivity for pathologic LNs in patients with clinically negative (cN0) head and neck cancer [15].

Radiomics is a novel promising tool for the extraction of additional information from medical images. There is evidence that deep learning (DL) in conjunction with radiomics may improve the detection of malignant lesions and the characterization of tumor tissue. Several studies have also shown its potential in differentiating benign from malignant lesions. For example, Zhang et al. [16] were able to distinguish Kimura disease from LN metastases by means of radiomics analysis on CECT scans. Kimura disease is a rare lymphoproliferative disease, which consists of painless subcutaneous soft tissue masses and goes along with an enlargement of LNs in the head and neck region, therefore easily being confused with LN metastases of an occult primary tumor. Seidler et al. [17] performed a texture analysis on cervical lymphadenopathy of dual-energy CT scans and could thereby differentiate between benign and pathologic nodules, which were either histologically proven metastases, lymphoma, or inflammatory LNs. Hence, radiomics based on quantitative measurement of relevant imaging features, such as the roundness and heterogeneity in density of LNs, would enable assessment of the likelihood of macro- and micrometastases in LNs. However, prior to performing radiomics analysis, an accurate segmentation of both small and large LNs is crucial. Head and neck CT scans contain about 300 LNs [18]. Thus, manual segmentations would be highly time-consuming and can in addition be subject to inter-reader variability. This is not feasible in clinical practice. Presumably to work around the time aspect, Seidler et al. did two-dimensional (2D) segmentations by drawing a region of interest around the largest diameter of the LNs in the axial plane and only examined the LNs’ texture on that single CT slice. By doing so, they potentially missed out further information on other slides, which could have been taken into account by performing volumetric LN segmentations prior to the DL analyses [19–23].

Therefore, this study aimed to develop an AI model for automatic localization and volumetric segmentation of cervical LNs on CECT scans of the head and neck region.

## Methods

### CT imaging selection and dataset

Patient consent was waived due to the retrospective design of the study based on preexisting images by the Ethics Committee of the Faculty of Medicine, University of Cologne, reference number 19–1390/07.08.2019.

Our picture archiving and communication system (IMPAX EE, Agfa HealthCare, Bonn, Germany) was searched for CECT scans of the head and neck region between January and November 2017. Inclusion criteria were clinically indicated CT examinations of the head and neck region (covering the skull base to the lung apex) with venous contrast enhancement and patient age ≥ 18 years. We excluded unenhanced examinations and/or scans containing most severe imaging artifacts (*e.g.*, beam hardening caused by large metal hardware or dense intravascular contrast agent accumulation, *n* = 2), while less significant artifacts (e.g., due to dental implants) did not result in exclusion of the examination. Follow-up scans from the same patient were excluded as were scans for which no LNs ≥ 5 mm could be detected in the manual segmentation process (*n* = 4).

All scans were conducted supine in cranio-caudal direction after a bolus injection of 80 mL of iodinated contrast agent (Accupaque, 350 mg/mL; General Electric Healthcare, Chicago, USA) with an injection rate of 3.5 mL/s and a delay of 40 s after reaching a threshold value of 150 HU in the descending aorta. Scans were performed on a dual detector layer CT scanner (IQon, Philips Healthcare, Amsterdam, the Netherlands) with the following scan parameters: tube voltage 120 kVp, tube current modulation (reference mAs 129, software DoseRight 3D-DOM, Philips Healthcare), collimation 64 × 0.625 mm, rotation time 0.33 s, and pitch 1.23.

Conventional, polychromatic CT images were reconstructed using a hybrid iterative reconstruction algorithm (iDose 4, filter B, level 3, Philips Healthcare) and a matrix of 512 × 512 in the axial plane with a slice thickness of 0.5 mm or 1.0 mm and a section increment of 50%.

The collected CECT scans were then subdivided in a training, validation, and test dataset. The validation set was used to tune the algorithm and explore the influence of different augmentation strategies in the training.

### Manual lymph node segmentation

For training, validation, and test datasets, all cervical LNs with an SAD of ≥ 5 mm were volumetrically segmented by one radiologist with more than 2 years of experience in CT imaging and double-checked by a second independent radiologist with more than 15 years of experience in CT imaging using the three-dimensional (3D) multimodal tumor tracking tool MMTT (originally available in IntelliSpace Portal, Version 12, Philips Healthcare) implemented in a dedicated research platform (IntelliSpace Discovery, ISD, Version 3.0, Philips Healthcare).

### Neural network preprocessing, architecture, training, and data augmentation

The following describes the training setup and the network architecture. The resulting model for cervical head and neck LN segmentation was available as prototype in IntelliSpace Discovery (Version 3.0, Philips Healthcare).

#### Preprocessing

Before training the convolutional neural network (CNN), images were resampled to a homogeneous and isotropic resolution of 1 mm^3^. To enhance the soft-tissue contrast of the LNs, image intensities were normalized from the window/level 395/10 HU to the interval [− 3, 3]. This intensity window was determined automatically for whole-body LN localization by taking the mean and standard deviation of image intensities inside the LN segmentations and their direct neighborhoods. The obtained interval is close to the CT soft tissue window/level of 350/50 HU. In addition, images were cropped to the head and neck region in case a larger field of view was present. This was also done automatically by cropping the images to a minimum size of 200 × 200 × 250 mm, ensuring that all LN annotations were contained in this box and enlarging it when required.

#### Architecture

For the segmentation of the cervical LNs, a foveal neural network architecture was chosen as it showed good performance previously for LN segmentation [24] as well as other tasks [25].

**Fig. 1:**
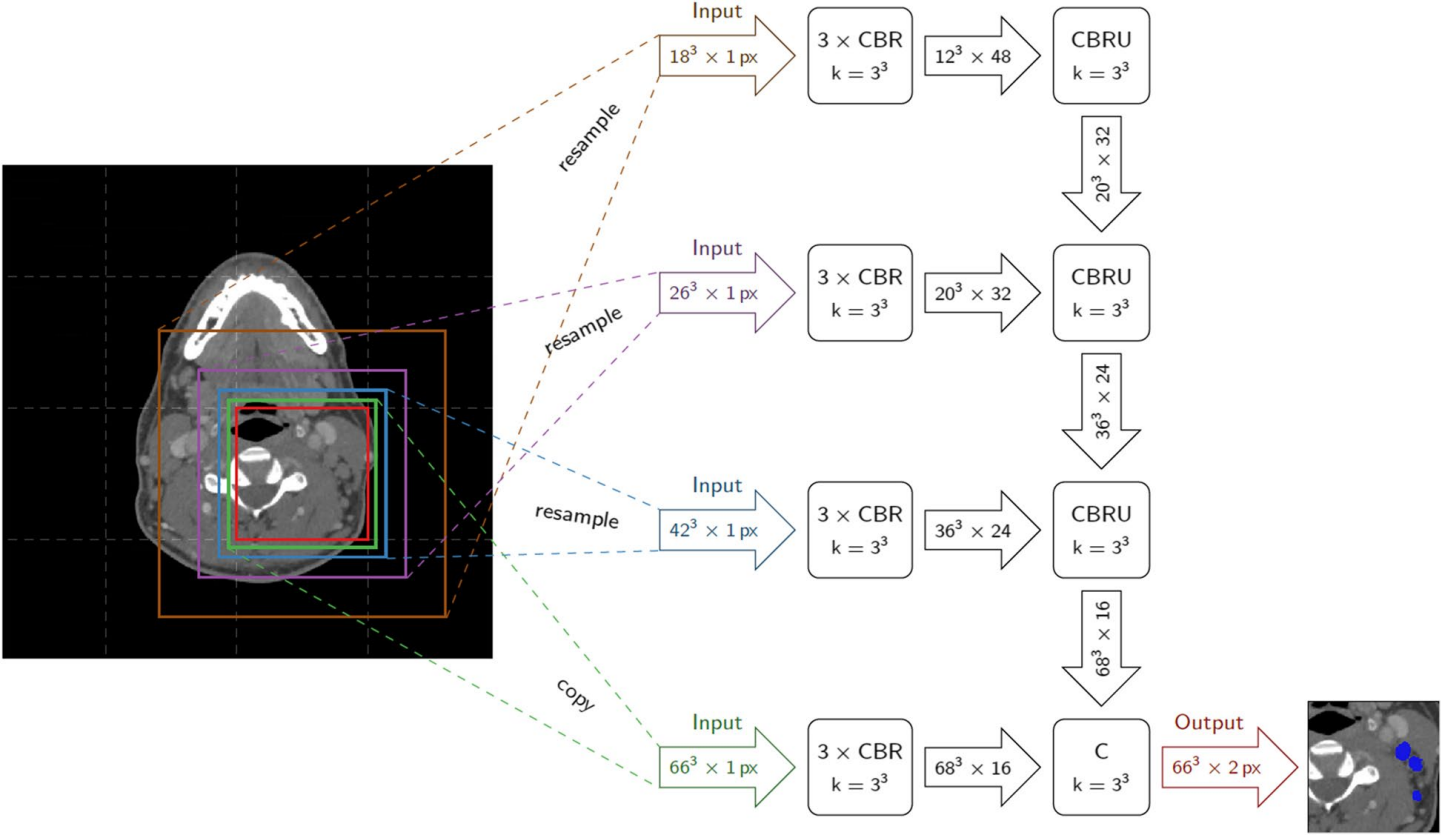
Illustration of the foveal neural network architecture. The network takes patches centered at the same location but with different size and resolution as input. It consists of several blocks of convolutional layers with kernel size of 3 × 3, batch normalization, and the rectified linear activation function (CBR). In the last column, the outputs of the CBR blocks are assembled and followed by an upsampling layer (CBRU). The final layer yields a probability map which is thresholded to obtain the final segmentation result. *C* Convolutional layer; *CBR* Convolutional layer, batch normalization layer, and rectified linear unit layer; *CBRU* Convolutional layer, batch normalization layer, rectified linear unit layer, and upsampling layers; *k* Kernel; *px* Pixel

The idea behind the foveal neural network is to use image patches with different resolutions as input to the CNN, thereby mimicking the behavior of the eye, which has the highest resolution at the *fovea centralis*. Small image patches have a finer resolution to provide a detailed view of the structures of interest, while larger patches have a coarse resolution better demonstrating the anatomical context as is visualized in Fig. 1. The output of the CNN is a map showing the probability of each voxel to represent LN tissue.

The employed network architecture has four resolution levels (see Fig. 1). Each resolution level extracts features from the corresponding patch at this resolution by three successive blocks of valid convolution with a kernel size of 3 × 3, followed by batch normalization and the rectified linear activation function: convolutional layer, batch normalization layer, and rectified linear unit layer (CBR). The outputs of the different resolution levels are assembled in the feature integration pathway via CBR blocks followed by an upsampling of the lower resolution output: CBR followed by upsampling layers (CBRU). Last, a softmax layer is applied to compute pseudo-probabilities representing the likelihood of each voxel belonging to an LN. By thresholding this output, the final segmentation mask is obtained.

For more information on the F-net architecture, we refer the reader to Brosch et al. [25].

#### Training

Training was performed using Python (3.8) with PyTorch (version 1.11.0). Random patches of size 72^3^ mm were drawn from the volumes and fed to the network. To correct for the class imbalance between foreground (*i.e.*, LN voxels) and background (*i.e.*, non-LN voxels), it was ensured that 50% of the patches contained foreground voxels. As loss, an equally weighted combination of the cross-entropy loss and the Dice coefficient loss [26] was chosen and optimized with the AdaDelta optimizer [27]. The CNN was trained for 2,000 epochs in minibatches where each minibatch contained 12 patches randomly drawn from different images.

#### Data augmentation

Three different augmentation strategies were explored during network training. All strategies were applied on the fly to the patches that were fed to the neural network. SimpleITK (version 2.02) was used for image manipulation. First, as basic data augmentation, random scaling and rotation of patches were applied with a maximal scaling factor of 1.2 and a maximum rotation of 10°. Parameters for the augmentation were drawn from a uniform distribution. Larger transformations showed a decline in performance and were therefore abandoned. Second, flipping along the sagittal plane with a probability of 0.5 was added to account for the symmetry of the head and neck. Finally, the strongest augmentation was explored by locally distorting the image patches using B-spline transformations. Here, a grid of 5 × 5 × 5 control points per patch was defined. Deformation parameters for the grid points were drawn from a normal distribution with a standard deviation of 0.2. The patch was transformed to the deformed grid using B-splines of order 1.

### Evaluation criteria

Prior to the evaluation, a connected component analysis was performed on the segmentation mask to separate the LNs. All connected components comprising less than 3^3^ voxels were excluded from the analysis as they were considered being too small. For these components, the SAD was clearly below the envisioned 5 mm.

After filtering out the very small localizations, the performance of the CNN was assessed using the following metrics: first, the percentage of correctly identified LNs (localization rate, LR) and the average number of false positives (FPs) per volume were assessed. Second, the global Dice was computed per CT scan taking only the true positives (TPs) into account to get a measure of the segmentation accuracy.

When analyzing individual LNs, we refrained from computing the Dice coefficient and instead computed the segmentation sensitivity of each ground-truth LN, *i.e.*, the ratio of correctly segmented LN tissue and LN volume. The reason for this is that we only had the connected components and not the individual LN masks from the network prediction. In case predicted LNs were touching, they would form one connected component and thus bias the Dice coefficient. For a more detailed evaluation, LNs were divided into SAD-dependent groups: 5–10 mm, ≥ 10 mm and ≥ 15 mm, according to RECIST 1.1 with LNs < 10 mm as physiological or unclear, ≥ 10 mm as suspected metastasis, and ≥ 15 mm as target lesions.

The evaluation metrics is reported on the training and validation data to assess the quality of the model and on the test dataset to explore how well the model performs on unseen data.

### Statistical analysis

Data regarding patients’ age, runtime, LR, and differences in SAD between FP and annotated LNs are presented as mean ± standard deviation.

Statistical analysis was performed using Python (3.7) with SciPy Stats package (version 1.7.3). Before the statistical analysis, the age information was grouped into five groups (< 40, 40–51, 53–59, 60–69, > 70 years) as well as the number of annotated LNs (< 7, 7–13, 14–20, 21–34, > 34 LNs). Due to the lack of normality of the data, a Kruskal–Wallis analysis was performed, considering a *p*-value 0.01 as threshold for significance. Furthermore, Pearson correlation was used to assess the correlation between SAD and global Dice. Point biserial correlation was used to assess if localization was correlated to SAD. A *t*-test was performed to evaluate the differences in SAD between FP and annotated LNs. We applied no correction for multiple testing.

## Results

### Study population

**Fig. 2:**
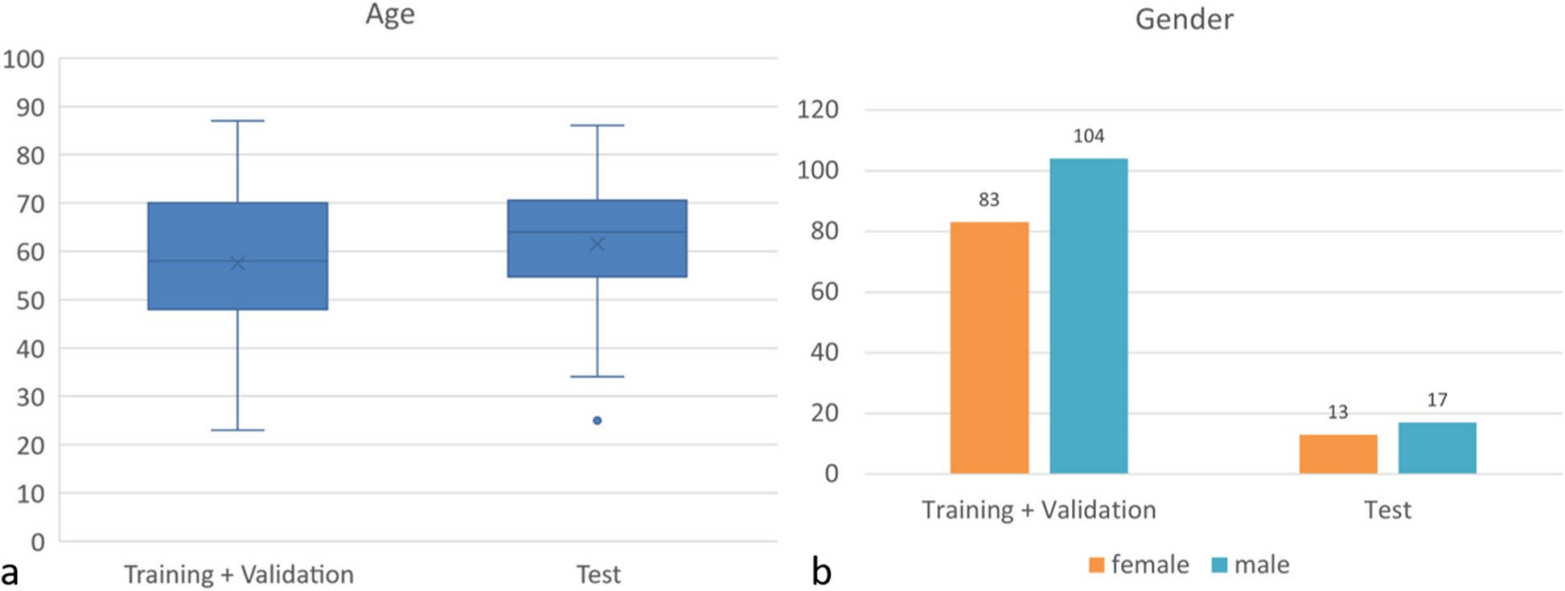
Gender and age distribution in the training plus validation and test dataset. Box and whiskers plot showing the age distribution in years on the training plus validation and test dataset (**a**) and bar chart showing the gender distribution in the training plus validation and test dataset (**b**)

In total, 217 CECT scans of the head and neck region from patients with various primary diseases were collected from our local database. Thirty of these scans with at least one clinically enlarged LN were put aside as test dataset. The remaining 187 CECT scans were randomly divided into independent scans for training (*n* = 150) and validation (*n* = 37, 1/5 of the data). In the training and validation dataset, 83 patients were female and 104 male, with an average age of 57.5 ± 15.5 years (females 58.1 ± 15.8, males 57.1 ± 15.1 years), while in the test dataset, 13 were female and 17 were male, with an average age of 61.5 years ± 14.9 years (females 58.7 ± 15.0 years, males 63.6 ± 15.0), as shown in Fig. 2.

**Fig. 3:**
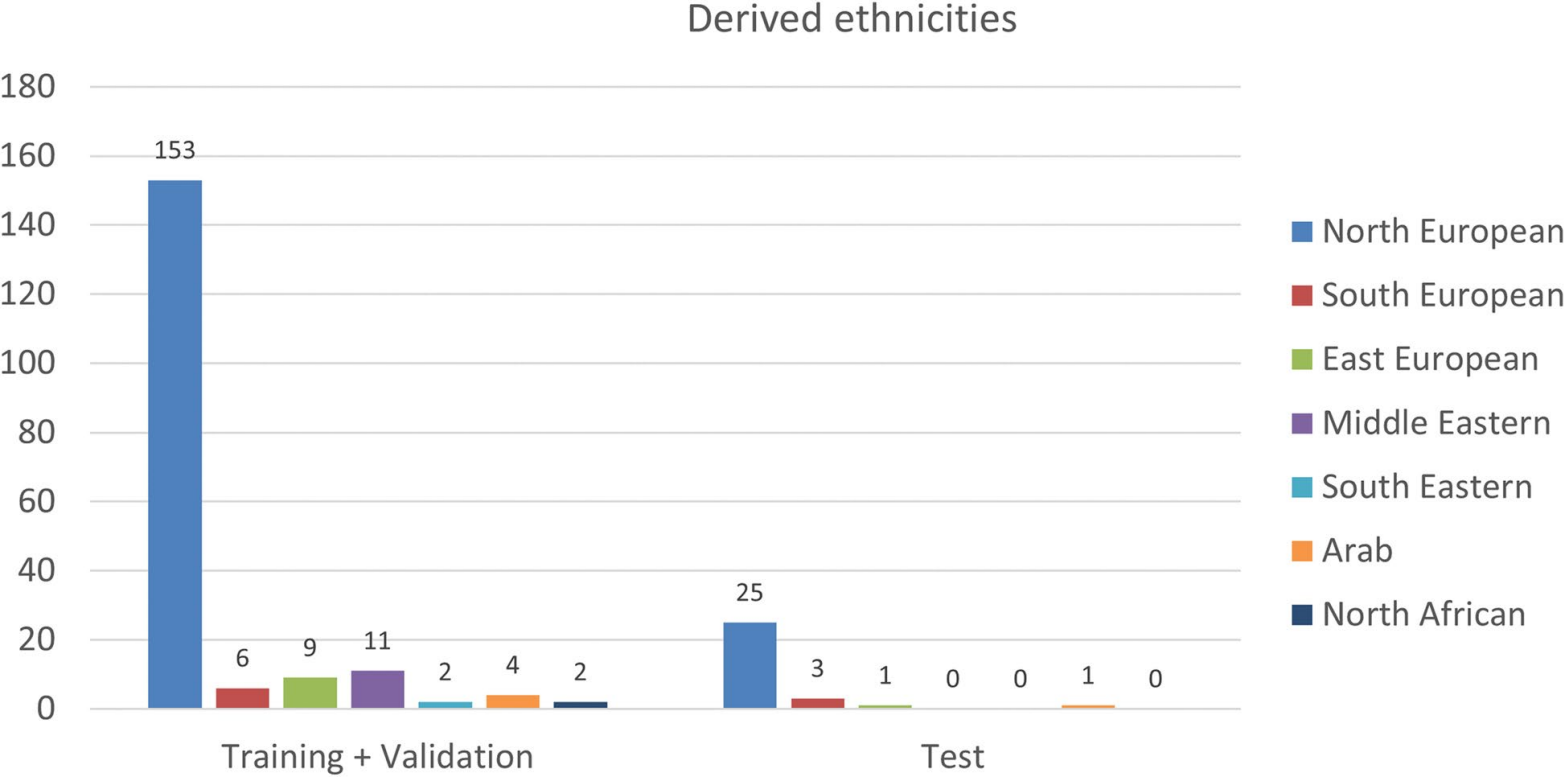
Derived ethnicities in the training plus validation and test dataset. Bar chart showing the derived ethnicities in the training plus validation and test dataset according to Flanagin et al. [28]

Since we only included scans from our local database, most patients were from the region around our institution, thus from a North European background. A further subdivision of the derived ethnicities in our dataset based on Flanagin et al. [28] is shown in Fig. 3.

**Fig. 4:**
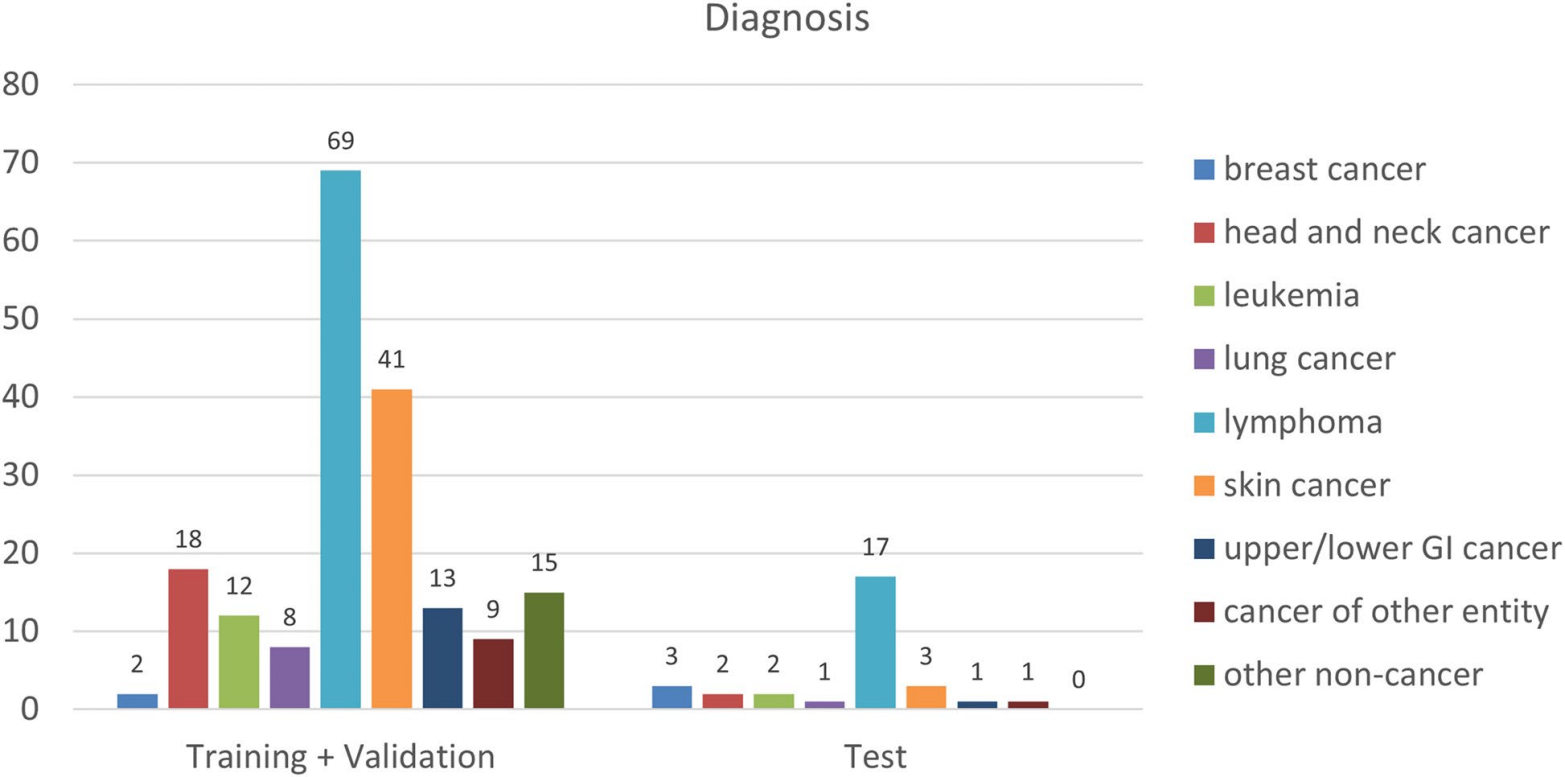
Distribution of pathologies amongst the training plus validation and test dataset. Bar chart showing the distribution of cancer and non-cancer diagnoses amongst the training plus validation and test dataset*.* *GI* Gastrointestinal tract

We included the scans independently of the underlying indication, resulting in a heterogeneous dataset with 202 cancer patients and 15 patients with a non-cancer indication for the CT scan as specified in Fig. 4. A further subdivision of the non-cancer indications as well as the types of cancer is shown in Supplementary Fig. S1.

### Manual lymph node segmentation

**Fig. 5:**
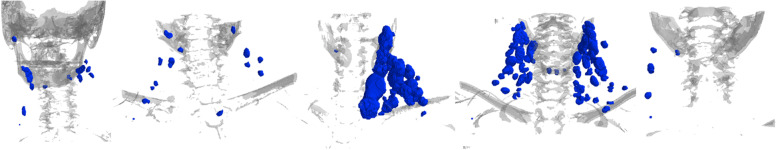
Example cases for manual lymph node segmentation. Renderings of the segmented lymph nodes on some exemplary training cases showing the variability in terms of number, size, and location of lymph nodes. Bones are rendered in gray for anatomical orientation

In the training and validation dataset, 3,656 LNs were segmented, yielding an average of 19.5 LNs per patient. The SAD was in a range between 2.68 and 43.8 mm. Examples for manually segmented LNs on some training cases are shown in Fig. 5. All segmented LNs were included in training, except for 570 inadvertently segmented LNs with an *SAD* < 5 mm. In the test dataset, 925 LNs with an SAD of ≥ 5 mm were segmented, yielding an average of 30.8 LNs per patient.

### Runtime

For a standard head-neck CT scan with a size of 512 × 512 × 400 voxels and an isotropic spacing of approximately 0.5 mm, the runtime accounts to 6.8 ± 0.1 s (average over 10 runs) on a nvidia GeForce GTX 1080 graphics processing unit. The time needed for manual segmentations strongly depends on the size of the scan and LN involvement. Here, scans with bulky disease pose the strongest challenge. While the effort of manually segmenting LNs in patients with mild LN involvement ranged from 30 min to 3 h, it could take up to several hours in patients with bulky disease such as CLL.

### Network performance in training and validation

The use of different augmentation strategies resulted in a Dice coefficient at training of 0.83 to 0.85 and at validation of 0.70 to 0.76 over all patients. Detailed results of the different augmentation strategies, as described in the section “data augmentation,” are shown in Table 1. Stronger augmentation led to a better generalization of the models, *i.e.*, while the performance degraded with stronger augmentation on the training data, it improved on the validation data especially with respect to the Dice. Overall, an average LR per volume of 78% was achieved on the validation data with an average Dice of 0.76. The FP rate also increased with stronger augmentation. Many of these “false positives” were either LNs which were rather small and were therefore not annotated or were located in the adjacent mediastinal region (see also below the “False-positive review” section for a detailed evaluation in the test data).

**Table 1:** Performance of the networks trained with different augmentation strategies on the training and validation data

Augmentation strategy	Training			Validation		
	LR (%)	FPs/V	DSC	LR (%)	FPs/V	DSC
Affine	88.5	1.4	0.85	65.5	5.4	0.70
Affine + mirror	86.0	3.9	0.84	72.6	7.6	0.73
Affine + B-spline	84.1	3.9	0.84	71.2	7.6	0.72
Affine + mirror + B-spline	86.8	7.8	0.83	78.0	10.7	0.76

Shown are the average localization rate per volume in percent (LR), the average false positives per volume (FP/V), and the average global Dice similarity coefficient (DSC) per volume taking only the true positives into account

### Network performance on the test dataset

**Fig. 6:**
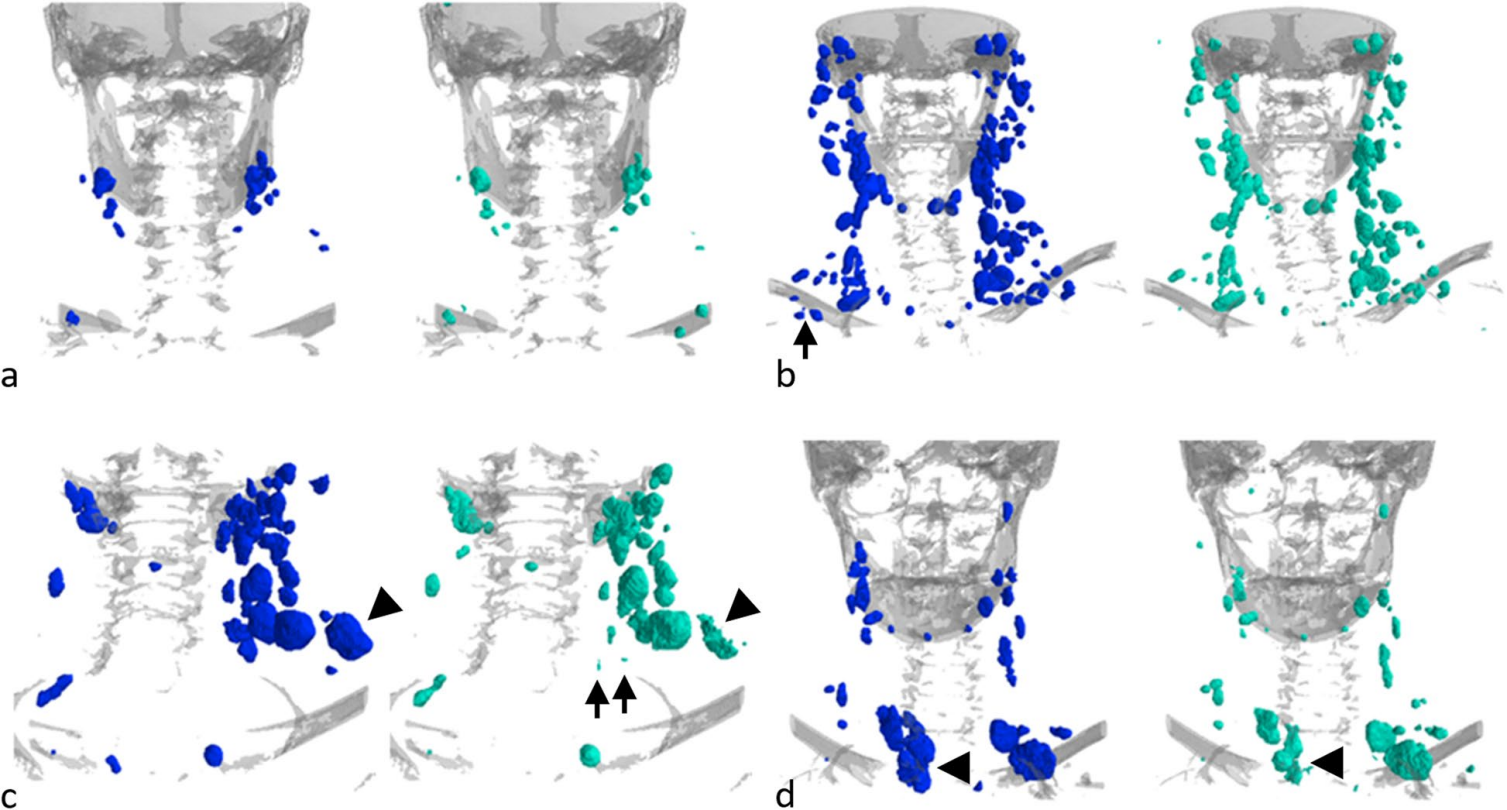
Exemplary renderings of the lymph node segmentations for four patients. Renderings of the lymph node segmentations for four patients (**a**–**d**) showing the manual annotations (left, blue) and the predicted segmentations (right, green). Bones are rendered in gray for anatomical orientation. Overall, a good agreement between ground truth and prediction was achieved. Some subclavicular lymph nodes in the mediastinum (**b**, arrow) and some very small lymph nodes (**c**, arrow) were segmented by the model, which had not been manually annotated (see the section “False-positive review”). For some of the larger lymph nodes (**c**, **d**, arrowhead), segmentation accuracy could be improved

Visual impressions of the predicted segmentations in comparison with the manual segmentations for exemplary cases from the test dataset can been seen in Fig. 6. The visual inspection shows good performance independent of location and size of LNs; only for a few very large LNs, the segmentation accuracy could be improved.

On the independent test dataset, the LR ranged from 66.4 to 81.1% and Dice between 0.66 and 0.71, depending on the augmentation strategy. In line with the results on the validation data, we also found an improvement with stronger augmentation, as given in Table 2. The LR improved from 66.4% with simple affine deformations to 81.1% using all tested augmentation strategies. The same applies to the segmentation accuracy, which improved from 0.66 to 0.71.

**Table 2:** Performance of the networks trained with different augmentation strategies on the test data

	Test		
	LR (%)	FPs/V	DSC
Affine	66.4	4.9	0.66
Affine + mirror	75.3	6.4	0.69
Affine + B-spline	73.3	6.1	0.69
Affine + mirror + B-spline	81.1	9.3	0.71

Shown are the average localization rate (LR) per scan in percent, the average false positives per volume (FP/V), and the average global Dice similarity coefficient (DSC) per scan taking only the true positives into account

**Fig. 7:**
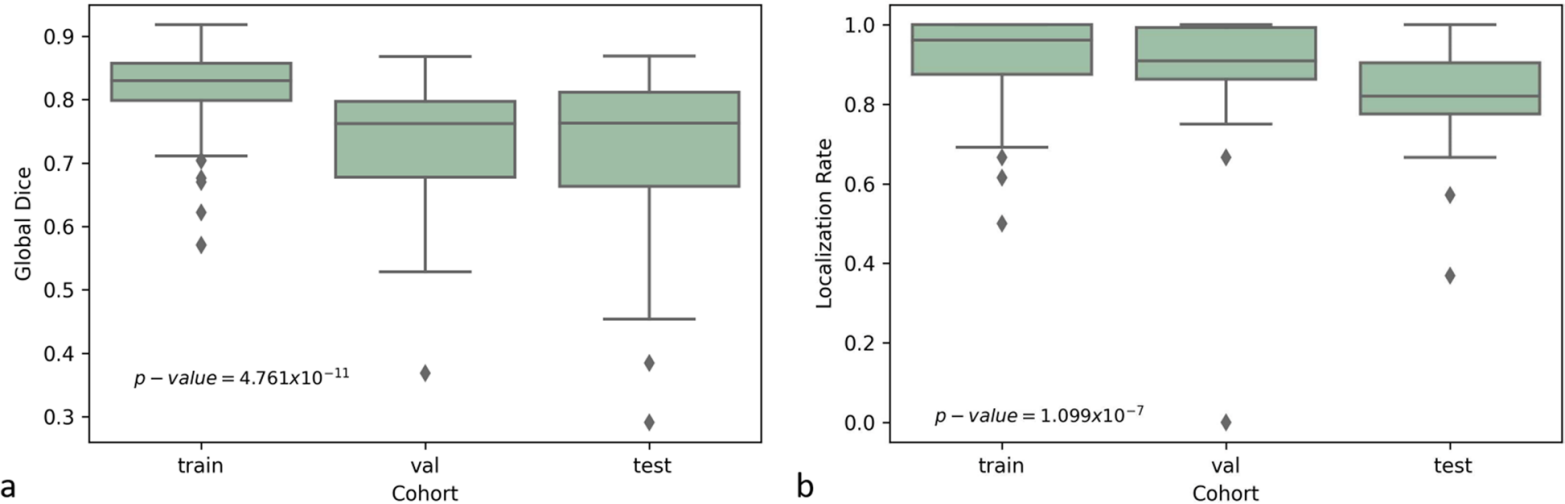
Box and whisker plots of the global Dice similarity score (**a**) and localization rate (**b**) for the training, validation, and test dataset

The statistical analysis (Kruskal–Wallis test) showed that for the variables global Dice and LR, there were no significant differences concerning sex (Dice, *p* = 0.095; LR, *p* = 0.050), age (Dice, *p* = 0.551; LR: *p* = 0.234), or the total number of annotated LNs (Dice, *p* = 0.910; LR, *p* = 0.022). For the global Dice, there were significant differences between the cohort groups (*p* = 4.671 × 10^−11^, Fig. 7a), and a more detailed analysis showed that there were significant differences between the training and test group (*p* = 1.201 × 10^−6^) as well as the training and validation group (*p* = 1.217 × 10^−7^), but not between the validation and test group (*p* = 0.920). For the LR, there were also significant differences between the cohort groups (*p* = 1.099 × 10^−7^, Fig. 7b), while there were significant differences (*p* = 0.001) for the LR between the validation (LR = 0.88 ± 0.17) and the test group (LR = 0.81 ± 0.12) as well as between the train (LR = 0.93 ± 0.09) and the test group (*p* = 3.987 × 10^−8^), but no statistically significant differences between LR in the train and the validation group (*p* = 0.032), as can be seen in Fig. 7b.

### Analysis of the false positives

For the FPs, Kruskal–Wallis analysis showed no significant differences concerning age (*p* = 0.273) and total number of annotated LNs (*p* = 0.304). However, there were significant differences concerning sex (*p* = 5.644 × 10^−5^). A more detailed analysis showed that these differences were only significant in the train group (*p* = 1.822 × 10^−6^), meaning that in the validation (*p* = 0.166) and test groups (*p* = 0.236), there were no significant differences between men and women.

There were also significant differences between the different groups (*p* = 2.541 × 10^−11^). A detailed analysis showed significant differences between the test group and train group (*p* = 7.585 × 10^−11^) as well as the test group and validation group (*p* = 2.975 × 10^−9^), but not between the train and validation (*p* = 0.071). In the test group, there were significantly less FPs (< 10 FPs/scan) than in the other two groups (> 20 FPs/scan).

### False-positive review

For the best-performing model, a review of the FPs was performed on the test dataset. For this analysis, all connected findings of the prediction were taken into account regardless of their size. The aim was to get a good impression of what the CNN considers to be an LN.

**Fig. 8:**
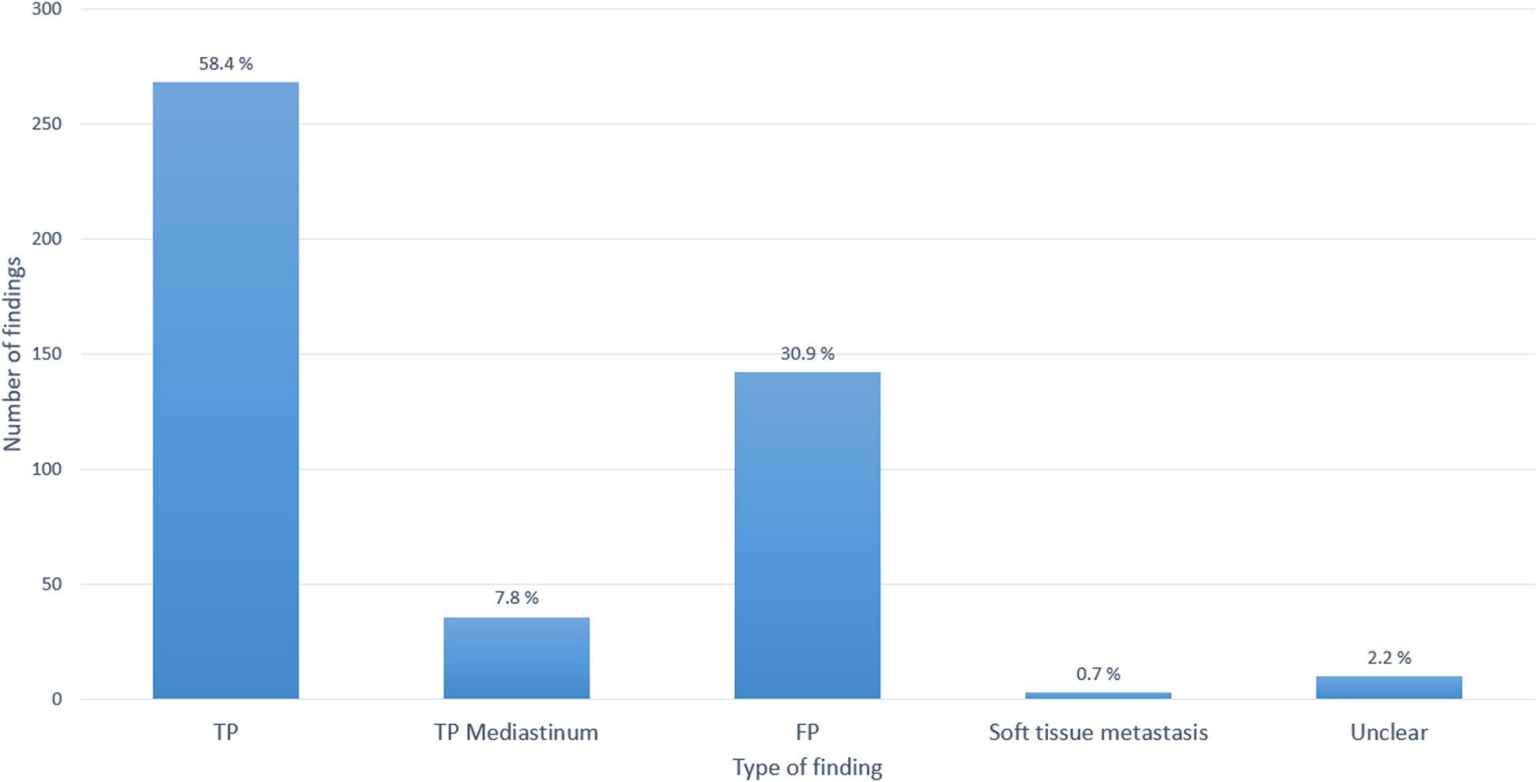
Bar chart showing the distribution of the different types of initially marked false positives. *FP* False positive, *TP* True positive, *TP* *Mediastinum* True positive in the mediastinum

A total of 459 findings were analyzed. We found that the vast majority (*n* = 268) of these findings were true-positive LNs, which had not been manually annotated due to their small size < 5 mm (Fig. 6c), and 36 of the findings were true LN but were located in the mediastinal region (Fig. 6b), for which no manual segmentations were available. For another 13 findings, it was not fully clear if they represented a real LN or soft or scar tissue. Consequently, the remaining 142 findings were considered as FPs. In these cases, the model marked other round-shaped structures such as soft tissue, muscles (*e.g.*, the scalene muscles), or vessels. A diagram illustrating these results is shown in Fig. 8.

After exclusion of the identified TPs, the average number of FPs initially reported in Table 2 reduced from 9.3 to 2.4 FPs on average.

In line, the *t*-test showed a significant difference between the SAD of the initially reported FPs with a mean SAD of 3.2 mm, thus below 5 mm, compared to the annotated LNs (*p* < 0.001).

### Network performance relative to lymph node size

In the previous sections, we evaluated all metrics on a CT scan level. In this section, we want to have a closer look and evaluate the performance on a per LN level. Please note that in Tables 1 and 2, the average LR per CT scan and not per LN was reported.

Overall, regarding the localization of individual LNs, our model was able to identify 93.3% of the manually segmented LNs in the training dataset (Table 3). Moreover, LRs were analyzed for subgroups of smaller LNs with an SAD between 5 and 10 mm and enlarged LNs with an SAD of ≥ 10 mm and ≥ 15 mm. In the training dataset, the LR reached similar results for the three subgroups with 93.1% for LNs with an SAD of 5–10 mm, 94.9% for LNs with an SAD of ≥ 10 mm, and 92.7% for LNs with an SAD ≥ 15 mm. In the validation dataset, the model detected 92.1% of LNs with an SAD between 5 and 10 mm and 95.9% of LNs with an SAD ≥ 10 mm. When looking at the subgroup of LNs with an SAD ≥ 15 mm in the validation dataset, it detected 87.5%. However, it is of note that this size group consisted of eight LNs only out of which the model detected seven LNs and missed one.

**Table 3:** Lymph node localization rate according to dataset and lymph node size

Dataset	5–10 mm	≥ 10 mm	≥ 15 mm	Overall
Training	93.1 2,008/2,157	94.9 (225/237)	92.7 (38/41)	93.3 2,233/2,394
Validation	92.1 (592/643)	95.9 (47/49)	87.5 (7/8)	92.3 (639/692)
Test	84.4 (691/819)	96.2 (102/106)	95.0 (19/20)	85.7 (793/925)

Shown are the localization rate in the training, validation, and test dataset according to four lymph node size groups as well as the absolute numbers of LNs detected by the AI algorithm compared to the absolute number of manually segmented LNs

Regarding the performance in the independent test dataset, our model reached an overall LR of 85.7%. When we examined the individual size groups in the test dataset, an LR of only 84.4% was reached for the smaller LNs. However, for the enlarged LNs with an SAD of ≥ 10 mm and ≥ 15 mm, the LR was 96.2% and 95.0%, respectively.

To assess the segmentation quality, we computed the sensitivity, *i.e.*, ratio of correctly segmented LN tissue (see Table 4). As can be seen there, the segmentation accuracy declined for larger LNs. While LNs with an SAD of 5–10 mm had a sensitivity of 0.77 on average on the test set, it reduced to 0.64 for enlarged LNs (SAD ≥ 10 mm) and accounts to 0.47 for the subgroup of even larger LNs with an SAD ≥ 15 mm. However, the sample size was rather low, and statistical analysis showed that there was no correlation between the SAD and sensitivity (*r* = 0.018, Pearson correlation), which points out that the AI segmentation model is working independently of the size of the LNs. Point biserial correlation between sensitivity and LR was very low (*rpb* =-0.026), meaning that there is no correlation between LN size and LR.

**Table 4:** Sensitivity of lymph node localization in the different datasets in relation to lymph node size

Dataset	5–10 mm	≥ 10 mm	≥ 15 mm	Overall
Training	0.74 ± 0.24	0.77 ± 0.21	0.83 ± 0.17	0.74 ± 0.24
Validation	0.72 ± 0.26	0.61 ± 0.32	0.52 ± 0.29	0.70 ± 0.28
Test	0.77 ± 0.22	0.64 ± 0.28	0.47 ± 0.26	0.75 ± 0.23

Shown is the sensitivity of lymph node segmentation performed by the AI model compared to manual segmentations in the training, validation, and test dataset according to four lymph node size groups. Data are given as mean ± standard deviation

**Fig. 9:**
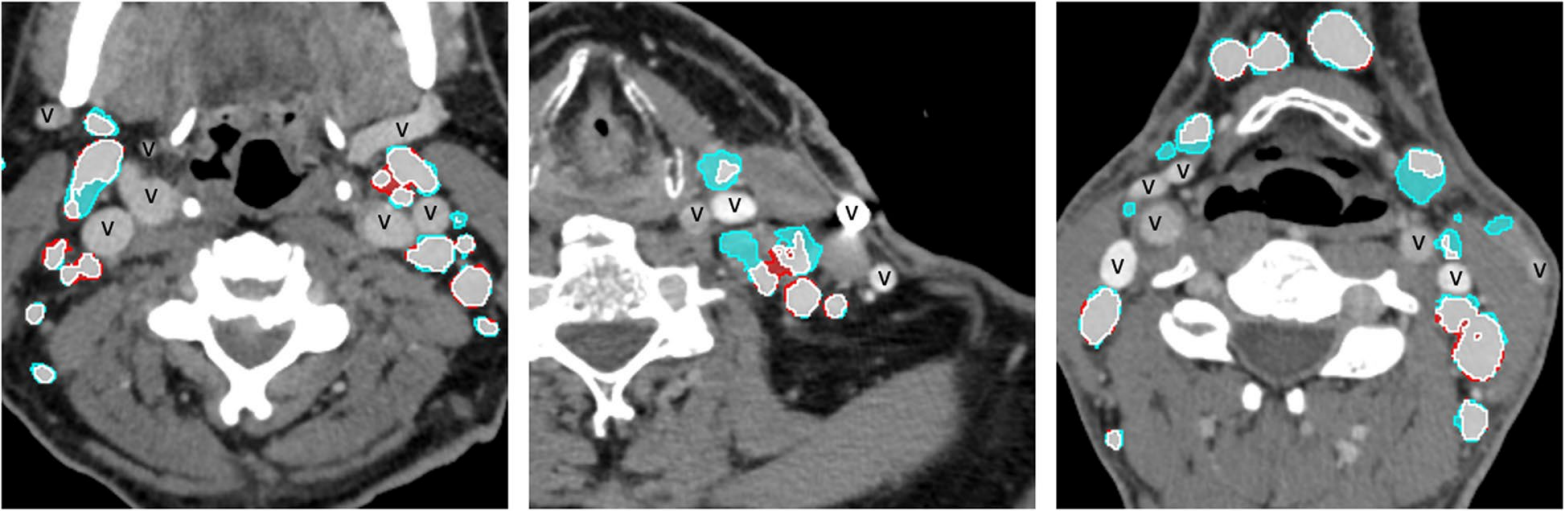
Overlay of segmentations in example cases which contain both good and poor artificial intelligence-conducted segmentations. Areas segmented both manually and by the AI model are marked in white, areas segment manually only are marked in cyan, and areas segmented only by the AI model are marked in red. To avoid confusion of axially cut vessels and LNs, vessels are marked with “v.”

Figure 9 shows that although some LNs were only partially segmented, in many cases, the LN segmentation was quite accurate.

## Discussion

In cancer management, it is essential to determine TNM status for therapy decisions, which thus directly affects the patient’s outcome. Assessment of nodal burden is often based on CECT, which allows accurate localization and measurement of LNs in the head and neck and facilitates evaluation of change over time. Complete determination of N status through thorough evaluation of a CECT scan is a very time-consuming task for radiologists in clinical routine and is subject to considerable inter-reader variabilities. A volumetric segmentation of LNs should enhance clinical care. It would also enable the development and application of advanced tools, such as radiomics, which may prove useful for further characterization of LNs, for example, to estimate the probability of active metastatic disease and the degree of therapeutic response.

Here, we aimed to develop a 3D DL model for robust LN localization and 3D segmentation in CECT scans of the head and neck region. Our AI model performed well and reached an overall LR of 85.7% on the independent test dataset compared to the manual segmentations. When comparing enlarged to non-enlarged LNs in the test dataset, the LR of 96.2% was even higher for the clinically suspicious LNs with an SAD ≥ 10 mm, which are considered worrisome for metastatic involvement in solid cancers according to RECIST 1.1 [6]. For target lesions according to RECIST 1.1, thus LNs with an SAD ≥ 15 mm, LR was 95.0%. Here, the slightly lower LR might be due to the small number of 20 LNs in that size group, out of which the model detected 19 and missed one. Since smaller LNs can also harbor micrometastases, we also analyzed the performance of our model for LNs with an SAD between 5 and 10 mm, for which the LR was 84.4%. Regarding segmentation accuracy, the model reached a Dice of 0.71 when compared to the manual segmentations. When looking at sensitivity of single LN segmentations, the algorithm showed a decline from 0.77 on average for LNs with an SAD between 5 and 10 mm to 0.64 for LNs ≥ 10 mm and 0.47 for LNs ≥ 15 mm. However, statistical analysis showed no correlation between SAD and sensitivity, so these findings might be due to the small number of cases in these subgroups.

For LN algorithm development, 2D approaches have been published based on the measurement of the axial SAD as used in the classification systems mentioned above [29, 30]. However, Mueller et al. [31] found that in the head and neck region, the 2D segmentation method tends to overestimate the LN volume. Using a 3D approach based on the perimeter, they found a closer correlation to the true LN volume determined after neck dissection.

In the head and neck region, only few prior investigations have been published for automatic LN segmentation, mostly relying on an atlas-based approach [32, 33]. One DL-based study concentrated on LN metastasis from thyroid cancer [34], and another included patients with oral squamous cell carcinoma [35]. In contrast, we included patients with various pathologies as well as normal nodal status to ensure a heterogeneous cohort for training and testing and to reflect broad applicability for clinical usage. Ariji et al. [35] focused on oral squamous cell carcinoma using the neural network “DetectNet.” They achieved a lower LR of 73% for metastatic LNs and 53% for nonmetastatic LNs in their test dataset compared to our results. Lee et al. [34] focused on thyroid cancer and evaluated eight deep CNN models. Of the eight models, ResNet50 was the best-performing model for the validation dataset, with an area under the ROC curve (AUROC) of 0.953. The sensitivity, specificity, and accuracy of the ResNet50 model were all 90.4%, respectively, in the test dataset. Here, it is important to note that the model was trained not only for localization but also for discrimination between benign and malignant LNs, making the sensitivity of their model more comparable to our localization of large LNs, where we achieved a LR of 96.2% in the test dataset.

Further investigation of the performance of our model, which was developed using a mixed patient cohort, could be performed for specific head and neck malignancies, such as thyroid cancer or oral squamous carcinoma. LN metastases from these tumors are often cystic or with central necrosis, making them distinct from those from other cancers, such as melanoma with more homogenous LN metastases [36]. The lower LR achieved by Ariji et al. who concentrated on oral squamous cell carcinoma and the higher LR in the specific training on metastatic lesions by the group working on thyroid cancer might point to this necessity.

Recently, Courot et al. [37] published a study similar to the present study, on the automatic detection of cervical lymphadenopathy in CT scans independently of the underlying malignancy. They combined three CNNs based on a three-dimensional version of an originally two-dimensional fully convolution network, U-Net, architecture. They reported a Dice of 0.63 for the evaluation dataset, which is lower than ours. However, they stated that the public dataset they used for training was poorly annotated due to time limits and consisted of only 117 CT scans. In contrast, we performed thorough manual segmentations for the complete dataset on a larger number of scans. Moreover, they only focused on lymphadenopathies and did not annotate non-enlarged LNs so that this model would not be suitable for research on characteristics for micrometastases.

To our knowledge, there is no other working group that developed an AI algorithm for volumetric segmentation of both enlarged and non-enlarged LNs in the head and neck region based on a heterogeneous cohort. A systematic literature search delivered no results of other studies on a mixed population but only investigations on cancer-specific cohorts such as the ones mentioned before.

When comparing the performance of our model for segmentation of the head and neck region to other body regions, we achieved a higher accuracy rate than reported by Iuga et al. [24] in the thorax where the LR was reported to be 69.9%. This might partially be due to a larger number of included CECT scans in our study with 187 CTs for training and validation, compared to 89 CTs in the study by Iuga et al. Moreover, both mediastinal and axillary LNs were localized by the thorax AI model, so the anatomical heterogeneity of these regions compared to the head and neck anatomy might have impacted segmentation accuracy.

In this work, we aimed to develop a robust and broadly usable AI model for potential clinical implementation. Therefore, we used a heterogeneous dataset and did not exclude scans with conglomerate LNs or bulky lesions even though this likely impacts sensitivity. Moreover, we did not exclude scans with metal artifacts from dental implants (apart from two images with severe artifacts). These artifacts are known to be a common challenge in analysis of CECT scans from head and neck cancer patients and are reported to be present in 73.6% of patients [38].

Several limitations of this study merit consideration. First, manual segmentation was performed by only one radiologist. This radiologist was well-trained, and the segmentations were double-checked by an experienced radiologist with more than 15 years of experience in a consensus approach. However, inter-reader agreement on LN segmentations has not been evaluated in this study and should therefore be further investigated in a subsequent study. Secondly, while we do have a large number of segmented LNs compared to other studies, all scans were acquired on the same scanner. Increasing the number of cases through a multi-scanner, multivendor and (international) multicenter approach would probably lead to an improvement in the LR and would make the model more generalizable in clinical practice. We used a local dataset for testing, whereas an external dataset might provide a better estimate of accuracy in a real-world clinical setting. Though our study did not specifically focus on different LN regions, this extension might be especially helpful to obtain an estimate of LR for underrepresented anatomic regions, *e.g.*, the retropharyngeal space, where LN metastases may be associated with a higher likelihood of distant metastasis [39]. Finally, we only segmented LNs with an SAD above 5 mm and did not consider smaller LNs, and our model showed a reduced performance on smaller nodules compared to larger ones. This could result in missing out clinically inconspicuous nodules, which can still harbor micrometastases and thus erroneously diagnosing a metastasized head and neck cancer as cN0. Here, it is again of note that measurement of LN size in the axial plane alone has proven not to be a sufficient criterion in determination of N status, but other aspects such as the LNs’ configuration, border, and shape need consideration as well, as reflected by Node-RADS version 1.0 [9].

The aim of our work was to provide a time-saving tool for a solid volumetric segmentation of both enlarged and non-enlarged LNs to enable AI-based research on these and potentially further characteristics to improve the accuracy of nodal status determination based on CECT scans. By reducing segmentation time from hours for manual segmentation to seconds for segmentations conducted by the AI model, it could not only serve as a base for research purposes but in a further step also enable integration of these potential findings into clinical practice.

Future work will aim at improving the LR of our algorithm. To secure segmentation accuracy for clinical applicability in terms of SAD measurements, also further improvement of the Dice and segmentation sensitivity are eligible. This might be achieved by increasing the training dataset, a time-consuming task, and the application of further data augmentation strategies. The use of AI-supported annotations with manual expert correction is a possible strategy. A larger training dataset would also facilitate the development and application of radiomics models for a variety of cancers affecting LNs of the head and neck region [40].

In conclusion, we developed and validated a 3D DL-based model that reliably localizes and volumetrically segments LNs in CECT scans of the head and neck region within seconds. Our approach can serve as a basis for further research on characteristics of nodal involvement especially in head and neck cancer patients but also other tumor entities and diseases affecting cervical LNs.

## Supplementary Information

**Additional file 1:** **Supplementary Fig. S1.** Specification of cancers and non-cancer pathologies for all scans in the training plus validation and test dataset.

## Data Availability

The datasets used and/or analyzed during the current study are available from the corresponding author on reasonable request.

## Abbreviations

2D: Two-dimensional
3D: Three-dimensional
AI: Artificial intelligence
CBR: Convolutional layer, batch normalization layer, and rectified linear unit layer
CBRU: Convolutional layer, batch normalization layer, rectified linear unit layer, and upsampling layers
CECT: Contrast-enhanced computed tomography
CNN: Convolutional neural network
CT: Computed tomography
DL: Deep learning
FP: False positive
LN: Lymph node
LR: Localization rate
Node-RADS: Lymph Node Reporting and Data System
RECIST: Response evaluation criteria in solid tumors
SAD: Short-axis diameter
TP: True positive
