# Supplementary material for: Automated localization and segmentation of cervical lymph nodes on contrast-enhanced CT using a 3D foveal fully convolutional neural network
Source: Eur Radiol Exp. 2023 Jul 28;7:45. doi: 10.1186/s41747-023-00360-x (PMC10382409; doi:10.1186/s41747-023-00360-x)

Automated localization and segmentation of cervical lymph nodes on contrast-enhanced CT using a 3D foveal fully convolutional neural network

ELECTRONIC SUPPLEMENTARY MATERIAL

Supplementary Fig. S1. Specification of cancers and non-cancer pathologies for all scans in the training plus validation and test dataset.

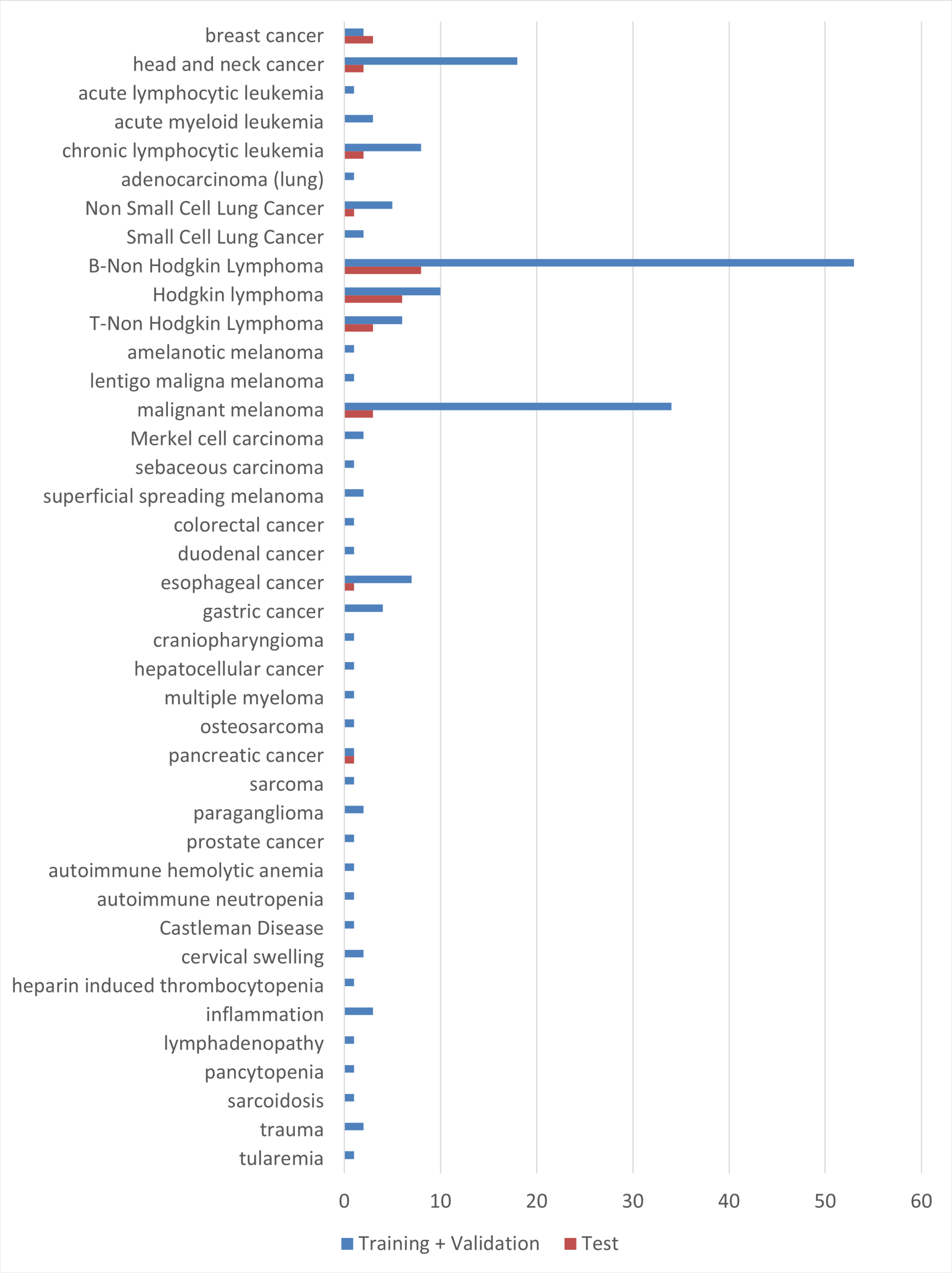

Supplement: Supplementary file 1 — Additional file 1: Supplementary Fig. S1. Specification of cancers and non-cancer pathologies for all scans in the training plus validation and test dataset. [file 41747_2023_360_MOESM1_ESM.pdf]
